# Supplementary figures and images for: Disentangling Semantic Composition and Semantic Association in the Left Temporal Lobe
Source: J Neurosci. 2021 Jul 28;41(30):6526–38. doi: 10.1523/JNEUROSCI.2317-20.2021 (PMC8318083; doi:10.1523/JNEUROSCI.2317-20.2021)

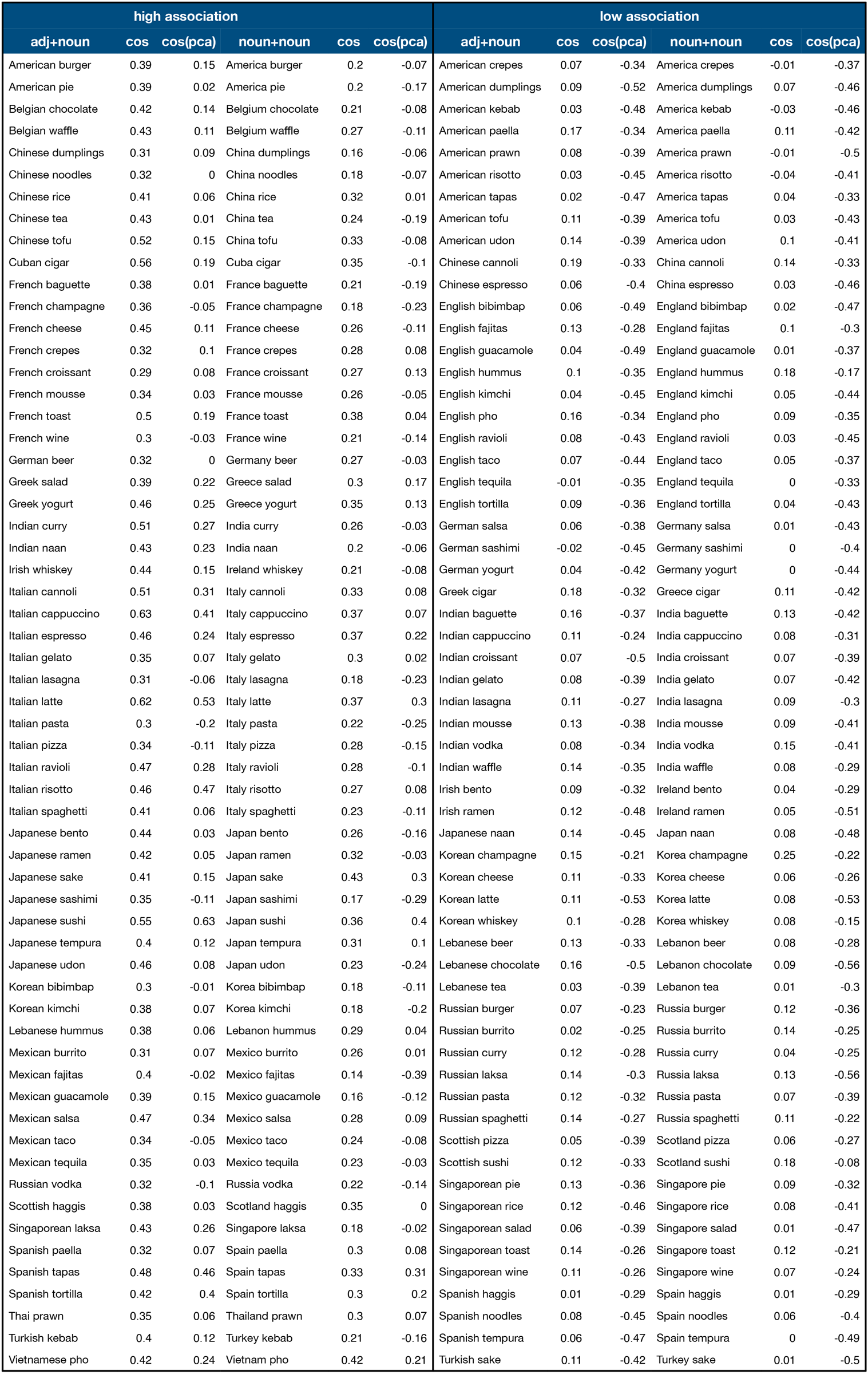

Supplement: Extended Data Figure 1-1 — Stimuli lists for experiment 1. Download Figure 1-1, TIF file. [file ns-JN-RM-2317-20-s01.tif]

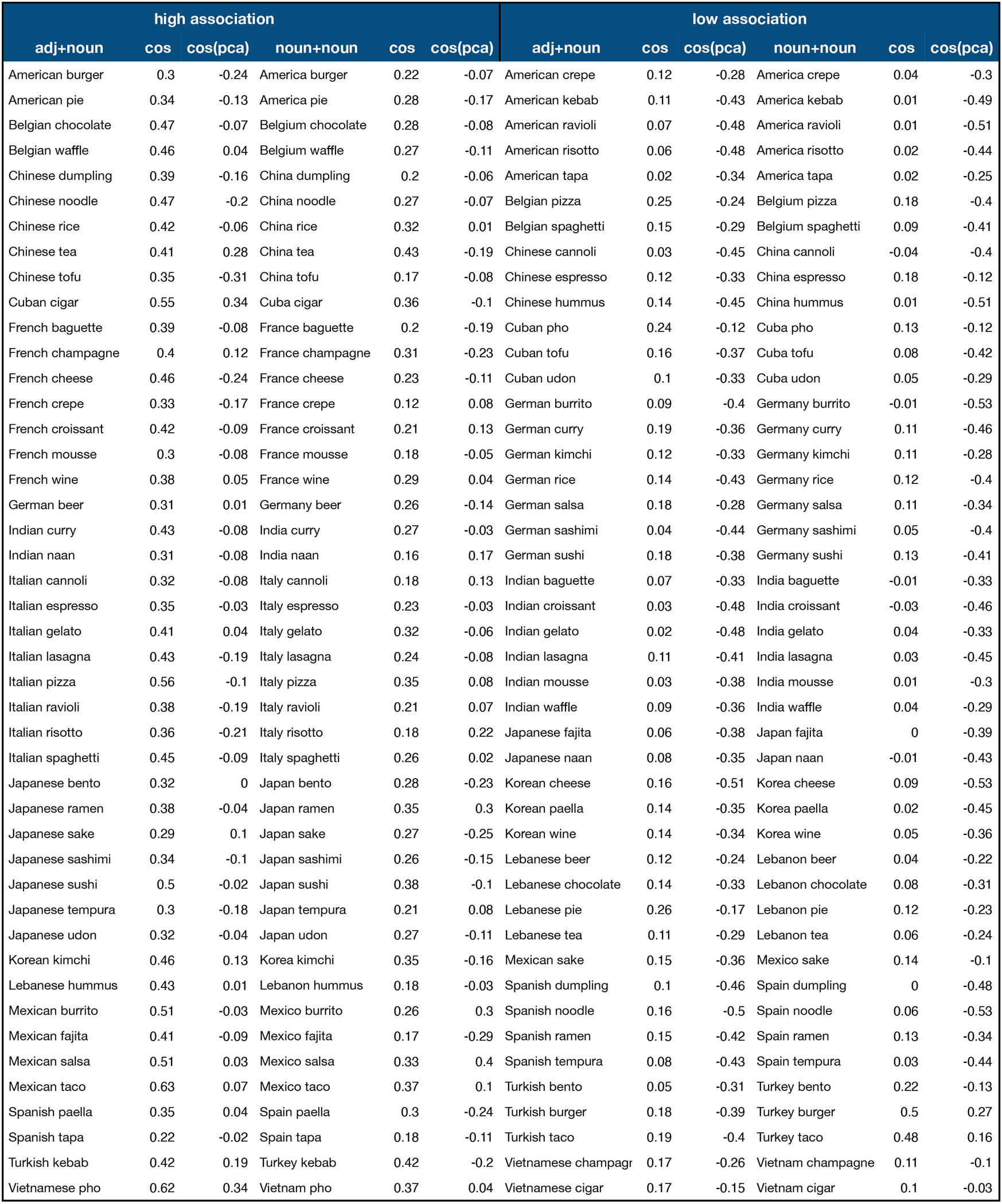

Supplement: Extended Data Figure 1-2 — Stimuli lists for experiment 2. Download Figure 1-2, TIF file. [file ns-JN-RM-2317-20-s02.tif]

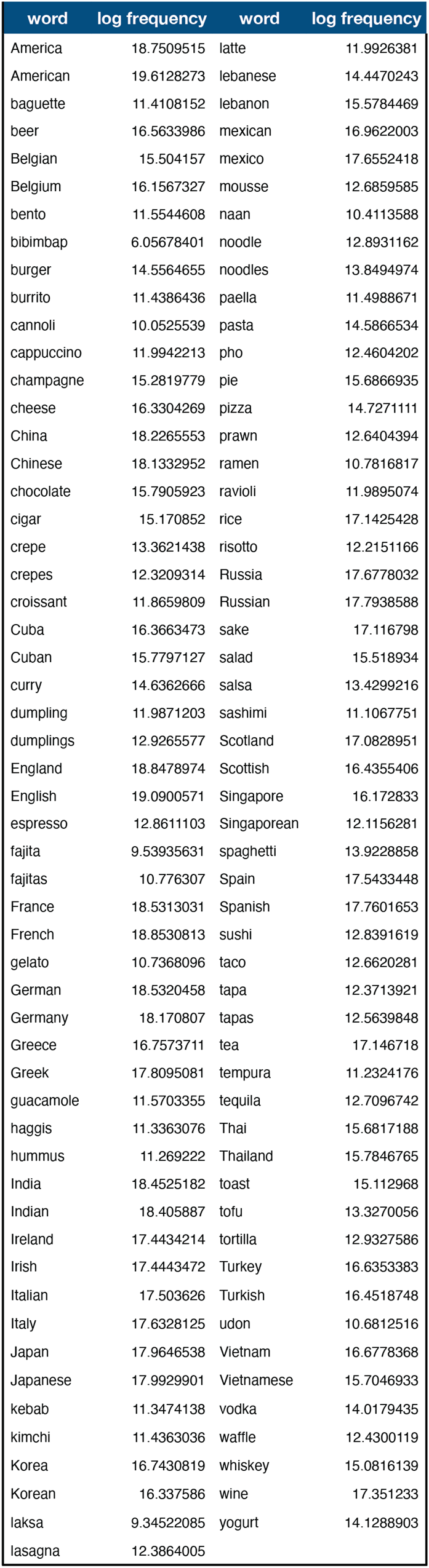

Supplement: Extended Data Figure 1-3 — Log frequency for all the words in experiments 1 and 2. Download Figure 1-3, TIF file. [file ns-JN-RM-2317-20-s03.tif]
